# Rapamycin Attenuates Anxiety and Depressive Behavior Induced by Helicobacter pylori in Association with Reduced Circulating Levels of Ghrelin

**DOI:** 10.1155/2022/2847672

**Published:** 2022-05-30

**Authors:** Jiageng Tian, Zeyu Wang, Yadi Ren, Yong Jiang, Ying Zhao, Man Li, Zhiguang Zhang

**Affiliations:** Department of Gastroenterology, The Second Hospital of Tianjin Medical University, Tianjin 300211, China

## Abstract

**Background:**

Helicobacter pylori (H. pylori) infection is closely associated with depression and development of neuroinflammation. The aim of this study is to explore the relationship between H. pylori, depression, and circulating levels of ghrelin.

**Methods:**

Mice were randomly divided into three groups: healthy control group (gavaged sterile saline and injected with saline, *n* = 8); H. pylori+saline group (gavaged H. pylori and injected with saline, *n* = 8); and H. pylori+rapa group (gavaged H. pylori and injected with rapamycin, *n* = 8). Open field test (OFT), sucrose preference test (SPT), forced swim test (FST), and tail suspension test (TST) were used for anxiety and depressive behavior test. Western blotting was utilized to assess mTOR, p-mTOR, and GSMD expression, and serum ghrelin levels were estimated using ELISA.

**Results:**

In the OFT, the control mice moved more and exhibited a increase in crossing number relative to the H. pylori+saline mice (all *P* < 0.05). Increased quantity of fecal boli can be indicative of increased anxiety and emotionality of the subject animal. H. pylori+saline mice exhibited an increase in fecal boli when compared to control mice and H. pylori+rapa mice (*P* < 0.05). H. pylori infected mice decreasing the expression of ghrelin. The protein levels of p-mTOR/mTOR in the gastric antrum mTOR signaling activation and low-level ghrelin in H. pylori-infect mice compared to those in control mice (all *P* <0.001). Compared with single H. pylori infection, mTOR inhibitors increased the ghrelin secretion of H. pylori infection to a certain extent (*P* < 0.05). The protein levels of GSDMD expression significantly increase in hippocampus of H. pylori-infected mice (*P* < 0.001). Rapamycin treatment inhibited expression of GSDMD in H. pylori-infected mice (*P* < 0.05).

**Conclusions:**

H. pylori infection is associated with increased expression of mTOR and decreased circulating levels of ghrelin. Elevated pyroptosis in the brain and anxiety- and depressed-like behaviors occur when ghrelin levels are suppressed.

## 1. Introduction

Helicobacter pylori (H. pylori) is a major contributor to gastritis, gastroduodenal ulcer, and gastric cancer worldwide [1, 2]. H. pylori causes chronic gastritis which lasts many years and even life-long without treatment [3]. H. pylori-induced gastric inflammation and atrophy are closely related with the neuroinflammation. Some researchers report a close relationship between Helicobacter pylori (H. pylori) infection and cognition and mental health [4, 5]. In particular, previous studies have shown that psychological factors are associated with H. pylori-induced neuroinflammation [4]. NLRP3 inflammasome-driven pathways have emerged as a crucial element in inflammatory or stress-related depression, according to a recent study [6].

Ghrelin is known as the only peripheral orexigenic peptide which is produced in the stomach. It is considered to be closely related with the brain-gut axis [7]. Ghrelin is secreted in response to acute stressors. And it is also persistently elevated in tandem with a variety of metabolic changes after exposure to chronic stress [8]. Recently, several studies have found that infection with Helicobacter pylori reduces ghrelin concentrations in both humans and rodents [9]. Ghrelin is secreted by X/A cells to which mTOR locates [10]. Meanwhile, changes of mTOR induced by Helicobacter pylori are significantly related to ghrelin secretion, and we hope to increase the basal level of ghrelin by lowering the expression of mTOR pathway. As a result, behavioral tests and hippocampal pyroptosis levels were employed to the boost ghrelin secretion, which were initiated by rapamycin induced inhibition of mTOR pathway.

The aim of this study was to evaluate the impact of H. pylori-induced pyroptosis in the hippocampus and its potential capacity to induce anxiety and depressive behavior of mice. The behavior and hippocampus GSDMD levels of Helicobacter pylori-infected mice were compared in relation to circulating levels of ghrelin.

## 2. Materials and Methods

### 2.1. H. pylori Strain

H. pylori strain (ATCC 43504), verified to be cytotoxin-associated gen A (CagA) and vacuolating cytotoxin A (VacA) positive, was obtained from the H. pylori Strain Pool (Shang, China). H. pylori contains 5% sheep blood at 37°C under microaerophilic air bag was used to culture the H. pylori strain for 48 h.

### 2.2. H. pylori Infection of Mice

Female C57 BL/6 mice of 4 to 6 weeks were used in this study. The mice were housed in the experimental animal facility with specific pathogen free (SPF) under standard care conditions. Animals were randomly divided into three groups. The mice of healthy control group, which were gavaged sterile saline and injected with saline (*n* = 8). For the mice of H. pylori+saline group (*n* = 8), H. pylori was gavaged and saline was injected. The mice of H. pylori+rapa group were gavaged H. pylori and inject with rapamycin (*n* = 8). After acclimatizatng for a week, the mice of H. pylori+saline group and H. pylori+rapa group were orally gavaged with 0.5 ml H. pylori suspension in H. pylori (1 × 10^9^ CFU/ml) daily for 5 days, whereas the mice of healthy control group were only gavaged with sterile saline. H. pylori+rapa group mice were administered intraperitoneally with rapamycin 1 mg/kg for 10 consecutive days after 16 weeks of normal feeding. The healthy control group and H. pylori+saline group mice were only administered intraperitoneally with saline.

After the experiment, the mice were sacrificed by cervical dislocation under CO_2_ narcosis. Blood samples were acquired promptly through cardiac puncture, and tissue samples were collected from the hippocampus and gastric antrum. The gastric antrum tissues were used for H. pylori infection examination. Stomach tissue was used for rapid urease test and HE staining to detect Helicobacter pylori. And stomach tissue also used for detecting of mTOR expression. Serum of mice was used to detect ghrelin expression. The hippocampus was used to detect the expression of GSDMD. This study was authorized by the Experimental Animal Ethics Committee and conducted in accordance with the principles outlined under the Guide for the Care and Use of Laboratory Animals (NIH publication 8623, National Institutes of Health, Bethesda, MD, 1985).

### 2.3. Open Field Test (OFT)

OFT was performed after injection was completed. Mice was individually placed in a square arena of 50 × 50 × 30 cm, which was divided into the peripheral area and central (25 × 25 cm) square area. The center region crossing number, total running distance, feces number, and center duration were for 5 min by a camera 2 m above the box. The field was cleaned with 75% ethanol after each test. Mice was tested once for 5 min by a camera 2 m above the box; then the field was cleaned with 75% ethanol after each test to avoid odor interference.

### 2.4. Sucrose Preference Test (SPT)

Prior to the experiment, the mice were trained to adapt to 1% sucrose for 48 h. Each mouse was deprived fluid and food for 8 h and then allowed access to 1% sucrose and tap water for 1 h. Sucrose preference was calculated as [(sucrose consumed)/(sucrose consumed + water consumed)]∗100.

### 2.5. Forced Swim Test (FST) and Tail Suspension Test (TST)

**Figure 1:**
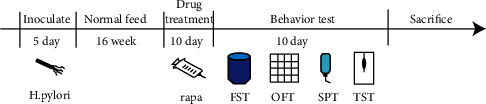
Flow diagram of the experiment.

After the sucrose preference test, FST and TST were performed to assess depression-like behavior. FST was conducted in a vertical transparent cylinder (30 cm in height × 15 cm in diameter) with the water to 30 cm. Mice were placed in the cylinder individually and recorded for 6 min. Mice were suspended by its tail in a box approximately 45 cm above the floor. Mice were kept immobile for an additional six minutes. The immobility period of the mice was measured after they were suspended for six minutes.  Figure 1 depicts the experiment's flow chart.

### 2.6. Evaluation Protocol

#### 2.6.1. Rapid Urease Test

The stomach is in a highly acidic environment which is difficult for the survival of bacteria. Helicobacter pylori produces urease, which locally breaks down urea and raises the pH of the surrounding environment. The rapid urease test is based on this characteristic of Helicobacter pylori and using urea and phenol red dissolved in water in proportion. When the added tissue contains the urease produced by Helicobacter pylori, the solution becomes alkaline and the color turns red. We dissolved 7 g of urea in 100 ml of distilled water in a conical flask. Then, 15 ml test tube was used to take 10 ml of distilled water to dissolve 40 mg of phenol red. Mix the above two solutions, and titrate the mixture with 0.1 N sulfuric acid to amber color (pH = 6.8). Use test tubes to aliquot the solution. The rapid urease test was utilized to detect the gastric antrum tissue of mice in experimental groups, and the rapid urease test turned red.

#### 2.6.2. HE Staining

Tissues were immersed in xylene I and xylene II until transparent. Then, tissues were dehydrated in 75%, 85%, and 95% ethanol solution. Cleared and dehydrated tissues block placed in melted paraffin. The embedded tissues were sectioned, baked, dewaxed, and hydrated. Hematoxylin staining and then eosin staining were performed. Finally, the sections were rinsed, dehydrated, cleared, mounted, and examined.

#### 2.6.3. Western Blotting Analyses

Total protein was obtained using Lysis buffer with added protease and phosphatase inhibitors from minced gastric antrum tissues. Then, the protein sample was separated by SDS-PAGE electrophoresis and then transferred to PVDF membranes (Millipore).

At room temperature, the membranes were blocked with 5% skimmed milk for 2 h. And then they were incubated overnight at 4°C with primary antibodies, including against mTOR (Proteintech, USA, 1 : 5000), p-mTOR (Proteintech, USA, 1 : 5000), GSDMD (Abcam, USA, 1 : 1000), and GADPH (Proteintech, USA, 1 : 10000). Following that, the membranes were washed and incubated with secondary antibodies for 2 h on a shaking table. Protein expression was quantified using grayscale values performed by ImageJ software and was standardized by a reference protein (GADPH).

#### 2.6.4. ELISA

The blood samples extracted from mice were used to measure the levels of serum ghrelin by ELISA. The ghrelin test kit was purchased from Xiamen Lunchangshuo Biotechnology Co., Ltd. (article number 20683). The whole procedure was carried out in accordance with the instructions provided by the kit.

### 2.7. Statistical Analysis

All data analysis was performed by GraphPad Prism 5.0. Data were presented as the mean ± standard deviation (SD). The results were analyzed using a one-way ANOVA and the Tukey honestly significant difference (HSD) multiple comparison test for comparisons among groups. Pearson's correlation analysis was used to compare differences between groups. *P* values < 0.05 were considered statistically significant.

## 3. Results

### 3.1. H. pylori Induced More Apparent Anxiety- and Depressive-Like Behaviours Than Control; the Use of Rapamycin Upregulated Ghrelin Secretion and Inhibited Anxiety- and Depression-Like Behavior in Mice

#### 3.1.1. SPT, TST, and FST

**Figure 2:**
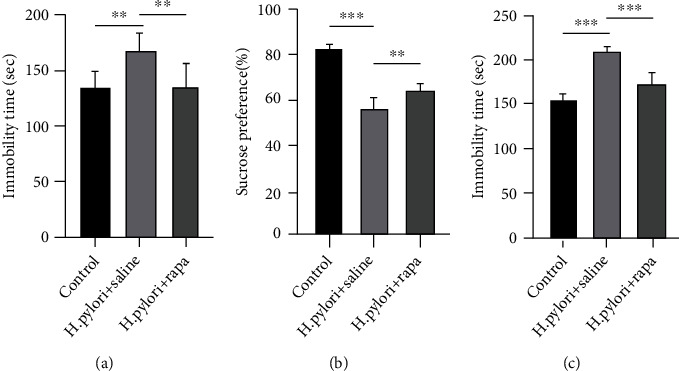
(a) FST, (b) SPT, and (c) TST of the control, H. pylori+saline, and H. pylori+rapa groups. ^∗^*P* < 0.05,  ^∗∗^*P* < 0.01, and^∗∗∗^*P* < 0.001 versus respective controls.

H. pylori reduced sucrose preference (*P* < 0.001, Figure 2(b)) and increased TST and FST immobility (*P* < 0.01, Figure 1(a) and 1(c)). Conversely, treatment with rapamycin reversed these effects in both assays for mice with H. pylori (*P* < 0.01 for sucrose preference, *P* < 0.01 for FST immobility, and *P* < 0.001 for TST immobility), indicating antidepressant properties of ghrelin.

#### 3.1.2. OFT

**Figure 3:**
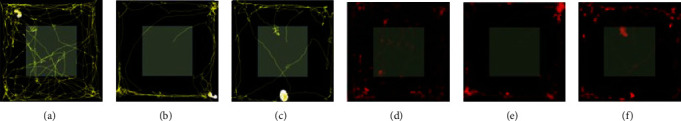
(a) Travel track and (d) spent time of control group; (b) travel track and (e) spent time of H. pylori+saline group; and (c) travel track and (f) spent time of H. pylori+rapa group.

**Figure 4:**
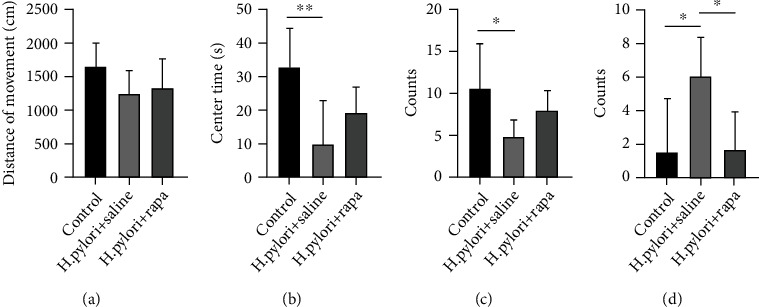
(a) Distance of movement, (b) center time, (c) crossing number, and (d) fecal feces counts of control, H. pylori+saline, and H. pylori+rapa groups. ^∗^*P* < 0.05,  ^∗∗^*P* < 0.01, and^∗∗∗^*P* < 0.001 versus respective controls.

Figure 3 represents the total distance traveled by the subject during 5 min time period of the test. The mice of control group track cross into the center portion of the maze at regular intervals while the H. pylori+saline mice track remains closely in proximity to the walls of the maze, indicating enhanced thigmotaxis or anxiety-related behavior. The contrast in red represents the time of mice spent in that area. The total distance traveled can represent the level of anxiety behavior. The distance of movement of the control mice was similar with H. pylori+saline mice. H. pylori+saline mice and H. pylori+rapa mice performed similarly in the OFM when total distance was measured (*P* > 0.05, Figure 4(a)). Time spent in the outer zones of the maze identified thigmotaxis or wall-hugging behavior and is indicative of anxiety-related behavior. The number of passes through the central area can represent the level of anxiety. Heightened number of central region crossing can be indicative of increased anxiety and emotionality of the subject animal. H. pylori+saline mice exhibited an decrease in crossing number and central time when compared to control mice (*P* < 0.05, Figures 4(b) and 4(c)). After mice were removed, the number of defecations or fecal excrements deposits was manually counted by the observer. An increase in the quantity of excrements can be a sign of the animal's increasing anxiousness and emotionality. H. pylori+saline mice exhibited an increase in fecal boli when compared to control mice and H. pylori+rapa mice (*P* < 0.05, Figure 4(d)).

#### 3.1.3. Confirmed Helicobacter pylori Infection

After rapid urease test and HE staining, we confirmed H. pylori infection in the gastric tissue of mice.

### 3.2. Effect of H. pylori Infection on mTOR Pathway Expression and Ghrelin Secretion in Gastric Antrum Cells

**Figure 5:**
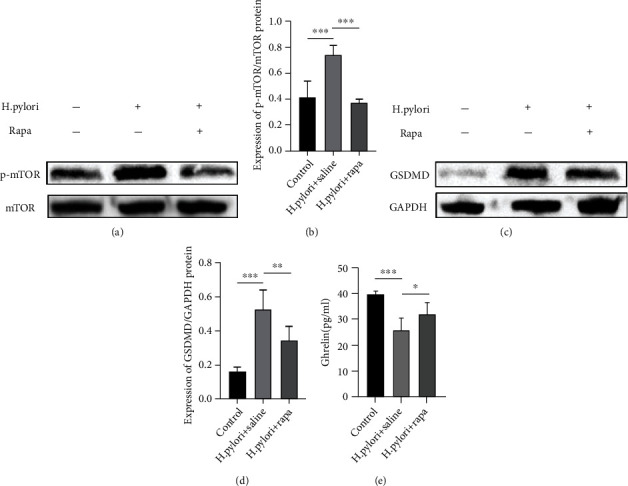
Western blot was performed to detect the protein levels of mTOR/p-mTOR (a) and GSDMD (c) expression. The protein expressions of p-mTOR (b) and GSDMD (d) were measured by densitometry and normalized to GAPDH. (e) The ELISA kits detected the levels of ghrelin. ^∗^*P* < 0.05,  ^∗∗^*P* < 0.01, and^∗∗∗^*P* < 0.001 versus respective controls.

The infection of the mice stomach by H. pylori decreases the ghrelin level in the plasma (Figure 5(e)). The protein levels of p-mTOR/mTOR in the gastric antrum are shown in Figures 5(a) and 5(b). Compared to those in control mice, the protein levels of p-mTOR /mTOR in the gastric antrum of H.pylori-infect mice showed significantly mTOR signaling activation and low-level ghrelin (all *P* < 0.05, Figures 5(c) and 5(e)). The ghrelin level is lower in the healthy control group (39.57 pg/ml) than in the H. pylori+saline group (25.40 pg/ml, *P* < 0.001).

### 3.3. Effect of Rapamycin on mTOR Pathway Expression and Ghrelin Secretion in Gastric Antrum Cells

To test whether the altered ghrelin expression because of mTOR pathway, we examined the expression of ghrelin in rapamycin treatment mice models. After correlation analysis, the result showed that the expression of mTOR is negative correlated with ghrelin level (*r* = −0.574, *P* = 0.013). Rapamycin effectively inhibits the mTOR pathway, and p-mTOR/mTOR decreased significantly (*P* < 0.001, Figures 5(a) and 5(b)). Compared with single H. pylori infection, mTOR inhibitors increased the ghrelin secretion of H. pylori infection to a certain extent (H. pylori+saline group (25.40 pg/ml) vs. H. pylori+rapa group (31.85 pg/ml), *P* < 0.05, Figure 5(e)).

### 3.4. The Level of GSDMD in the Hippocampus of Helicobacter pylori-Infected Mice Was Correlated with Anxiety- and Depression-Like Behavior; this Can Be Alleviated by Improving Ghrelin Secretion

In comparison to control mice, the protein levels of GSDMD expression significantly increase in the hippocampus of H. pylori infected mice (*P* < 0.001). Rapamycin treatment inhibited levels of GSDMD expression in H. pylori-infected mice (*P* = 0.002, Figures 5(c) and 5(d)). After correlation analysis, the result showed that the expression of GSDMD is negative correlated with ghrelin level (*r* = −0.863, *P* < 0.001).

## 4. Discussion

Numerous studies published recently have extensively examined the link between gastrointestinal diseases and depression [11]. The presence of HP infection has been implicated as a risk factor for depressed mood and mental illness [12]. Patients with a more severe form of Hp-associated atrophic gastritis had a higher risk of psychological discomfort and depression, although the explanation for this has not been determined conclusively [12]. During this investigation, mice were infected with H. pylori for a period of 19 weeks, which mimics a human chronic H. pylori infection. Our research demonstrated that H. pylori could induce anxiety-like behaviors, which were indicated in the results of OFT experiments. Increased time of immobility in FST and TST and reduction sucrose of consumption in SPT, which were confirmed H. pylori, could induce depression-like behaviors.

Cellular energy balance is maintained by the mTOR pathway. Ghrelin expression in the stomach mucosa and plasma may be reduced by increasing the activity of the mTOR pathway. And rapamycin, which inhibits mTOR signaling, stimulates the production of gastric ghrelin mRNA and raises plasma ghrelin levels, preventing ghrelin's circadian cycle [13]. The increased mTOR of gastric mucosa in H. pylori-infected patients and mice was largely dependent on cagA [14]. Infected C57 mice with H. pylori had higher mTOR expression and lower ghrelin release, according to this research. After inhibiting mTOR with rapamycin, we observed an increase in ghrelin release. Ghrelin secretion is lowered by H. pylori via mTOR, as shown in this study. Ghrelin is a hormone that is mostly produced in the stomach. It promotes growth hormone (GH) release by activating the GHSR [15]. Recent investigations demonstrate that hippocampal impairment is strongly associated with psychosis [16]. Some researches demonstrate that ghrelin has an effect on adult hippocampal neurogenesis. [17]. A study focus on rat lacking ghrelin receptor showed that ghrelin signal damaged induced the early onset decrement in hippocampal spine density and neurogenesis [18]. The abnormalities in animal behavior experiments induced by chronic unpredictable mild stress mice could be alleviated by chronic peripheral administration of ghrelin [19]. In accordance with our studies, rapamycin treatment on H. pylori mice ameliorated depression-like behaviors though ghrelin secretion increased. We concluded that downregulated ghrelin could be a reason for H. pylori causing anxiety- and depression-like behavior.

**Figure 6:**
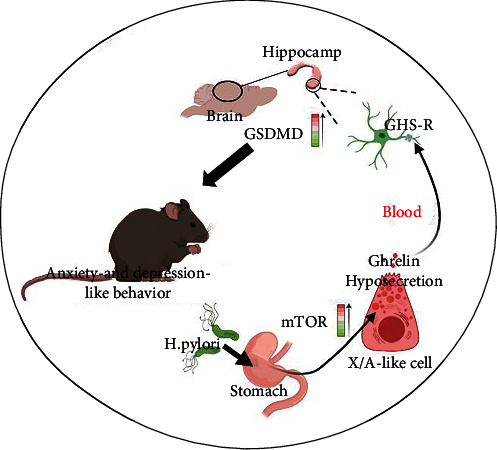
Schematic overview of H. pylori causes anxiety and depression-like behavior by ghrelin hyposecretion.

Previous research indicated that H. pylori infection induced systemic inflammation, which is associated with the development and progression of neurological diseases [20]. At present, LPS, the primary component of the outer membrane of Gram-negative bacteria, is commonly employed to induce depressive-like states in rodents [21, 22]. LPS-induced depressive-like states of rodents also exhibit NLRP3 inflammasome activation, most notably in the hippocampus [23, 24]. In immune cells, caspase-1-dependent pyroptotic cell death is owing to NLRP3 inflammasome activation [25]. Active caspase-1 cleaves Gasdermin D (GSDMD), which is a key in pyroptotic cell death. It has been reported that ghrelin attenuates brain injury by suppressing NLRP3 inflammasome activation [26]. Thus, we hypothesize that pyroptotic-induced neurological inflammation is the source of H. pylori-induced anxiety and depression. And downregulated ghrelin increased the risk of pyroptotic cell death in the hippocampus. The level of GSDMD in hippocampus expression verified our conjecture.  Figure 6 summarizes how H. pylori produces anxiety and depression-like behavior.

However, we must be aware of several possible drawbacks in our studies, because numerous studies have reported the protein pathways of the relevant mTOR and pyroptotic cell death. This study only detected the key proteins in mTOR and pyroptotic cell death pathways to explain the mechanism of anxiety and depression caused by H. pylori. Due to the absence of intervention on the upstream and downstream proteins of the pyroptotic cell death pathway in the hippocampus, it was not confirmed that pyroptotic cell death was responsible for H. pylori-induced anxiety and depressive behavior. We only conclude that boosting ghrelin expression can significantly decrease the level of pyroptotic cell death in the hippocampus and anxiety and depressive behaviors.

## 5. Conclusions

H. pylori inhibits ghrelin secretion by upregulating mTOR. Elevated pyroptotic-related neurological inflammation and anxiety- and depressed-like behaviors occur when ghrelin secretion inhibited in H. pylori infection.

## Data Availability

All figures submitted have been created by the authors, who confirm that the images are original with no duplication and have not been previously published in whole or in part.
